# Glycosaminoglycan-synthetic activity of neoplastic and non-neoplastic adipose tissues.

**DOI:** 10.1038/bjc.1980.261

**Published:** 1980-09

**Authors:** M. Sobue, K. Miura, K. Kataoka, K. Tsuji, J. Takeuchi

## Abstract

**Images:**


*Br. J. Cancer* (1980) **42**, 477

***Short Communication***

### GLYCOSAMINOGLYCAN-SYNTHETIC ACTIVITY OF NEOPLASTIC AND NON-NEOPLASTIC ADIPOSE TISSUES

M. SOBUE,* K. MIURA,† K. KATAOKA,‡ K. TSUJI* AND J. TAKEUCHI*

*From *the Department of Pathology, and †the Department of Surgery, School of Medicine, Fujita-Gakuen University, Toyoake, Aichi, and the ‡Department of Internal Medicine, Nagoya University School of Medicine, Nagoya, Japan*

Received 10 March 1980 Accepted 19 June 1980

IN WELL-DIFFERENTIATED liposarcoma there are commonly observed mucinous areas that have merged with a vascular tissue, mainly composed of small and large lipocytes of varying degrees of maturity. It was demonstrated by biochemical and histochemical investigations that the liposarcoma tissue contained glycosaminoglycans (GAG) consisting mainly of hyaluronic acid (Meyer, 1955; Winslow & Enzinger, 1960; Kaneko, 1974; Kindblom & Angervall, 1975; Filipe & Mackenzie, 1976) but a precise analysis of the synthetic activity of GAG in adipose tissue has not been performed. In the present study, we observed the GAG-synthetic activity of neoplastic and non-neoplastic adipose tissues by examining the incorporation of $^{3}H$-glucosamine or $^{35}SO_4$ into tissue GAG.

Each tissue used for analyses is shown in the Table.

Immediately after surgical excision, each tissue was cut into thin slices (1 mm thick) and the slices were incubated in the following medium: 10% dialysed calf serum (Research Institute for Microbial Diseases, Osaka University) in Eagle's minimal essential medium (F-12, GIBCO, Grand Island, N.Y., U.S.A.) containing 10 $\mu$Ci of $^{35}SO_4$/ml (sp. act. 0·33 Ci/mmol) or 10 $\mu$Ci of $^{3}H$-glucosamine (sp. act. 21 Ci/mmol). After 1h incubation at 37°C, the tissue slices were washed with 80% ethanol, embedded in paraffin and sectioned. The sections were stained with Alcian blue–haematoxylin and eosin, covered with photographic emulsion (SAKURA NR-H2, Konishiroku Photo Industries Co. Ltd, Tokyo) and an autoradiograph was made for the localization of labelled materials in the tissues.

Procedures to identify $^{35}S$- or $^{3}H$-labelled materials in the tissue segments after incubation (see above) were essentially the same as described in our previous papers (Takeuchi *et al.*, 1978; Sobue *et al.*, 1980). The labelled tissue segments were washed with 80% aqueous ethanol several times to remove free isotopes, and dried with acetone. After being weighed, the resulting dry powder was suspended in 0·3M NaOH and kept at 4°C overnight. It was then neutralized with 1M HCl, adjusted to pH 8·0 with 1M Tris-HCl buffer, and digested with pronase. Undigested materials in each tube were discarded by centrifugation at 3000 rev/min for 15 min. Glycosaminoglycans were precipitated from the supernatant by adding 3 volumes of 95% ethanol containing 1% potassium acetate. Ethanol precipitation was repeated 3 times, and the precipitate was washed with 80% ethanol, and acetone dried. The materials obtained were dissolved in 0·1 ml of water per mg dry weight of the tissue. An aliquot was taken to determine the uronic acid concentration

Correspondence to: Dr Mitsuko Sobue, Department of Pathology, School of Medicine, Fujita-Gakuen University, Toyoake, Aichi 470-11, Japan.

TABLE.—*Radioactivity of ³H-hyaluronic acid, ³H-sulphated glycosaminoglycans, ³⁵S-dermatan sulphate and ³⁵S-chondroitin sulphate in each tissue (ct/min/mg of defatted tissue)*

| Case | Age | Sex | Histological diagnosis | $^{3}$H-hyal-uronic acid | $^{3}$H-sul-phated GAG | $^{35}$S-der-matan sulphate | $^{35}$S-chon-droitin sulphate | U.A.* |
|---|---|---|---|---|---|---|---|---|
| 1a | 71 | M | Liposarcoma with myxoid area† | 4872 | 663 | 330 | 62 | 277–355 |
| 1b | 71 | M | Liposarcoma with myxoid area‡ | 2231 | 248 | 529 | 302 | 146–148 |
| | | | Subcutaneous adipose tissue (abdominal wall) | 40 | 50 | 113 | 94 | 7·3–7·4 |
| 2 | 18 | F | Lipoma (size of an egg) (subcutane, shoulder) | 172 | 211 | 256 | 63 | 23·2–25·6 |
| 3 | 71 | M | Lipoma (size of a fist) (subcutane, back) | 42 | 38 | 469 | 239 | 10·9–11·0 |
| 4 | 43 | M | Adipose tissue (omentum) | 20 | 43 | 289 | 64 | 11·2–11·8 |
| | | | Subcutaneous adipose tissue (abdominal wall) | 14 | 16 | 133 | 49 | 8·8–11·1 |
| 5 | 58 | M | Adipose tissue (omentum) | 20 | 31 | 110 | 125 | 10·4–11·0 |
| | | | Subcutaneous adipose tissue | 39 | 39 | 11 | 19 | 6·3–6·8 |

* The range of uronic acid content (nmol/mg defatted tissue) in 3 pieces of each tissue.
† Tumour tissue (4·3 kg) from the retroperitoneal space, and occupying the abdominal cavity.
‡ Recurrent tumour tissue (5·8 kg) extirpated about 8 months after the excisions of Case 1a.
After defatted tissue was treated with 0·3 N NaOH and digested with pronase, an aliquot of the resulting homogenate was subjected to a paper chromatography with butanol/acetic acid/0·5M ammonia (2:3:1 v/v) in which GAGs had little mobility, as described in our previous paper (Sobue *et al.*, 1980). About 80–90% of the radioactivity remaining at the origin of the paper chromatogram could be detected in the GAG fraction.

by the procedure of Bitter & Muir (1962). Each solution containing 10 nmol of uronic acid was spotted on a cellulose-acetate membrane and electrophoresed under the condition described in our previous paper (Sobue *et al.*, 1978). GAG components (hyaluronic acid, chondroitin sulphate, dermatan sulphate and heparan sulphate) were separated from each other by this procedure. The strips were stained with 0·2% Alcian blue in 0·1% acetic acid. Radioactivity incorporated into the individual component of glycosaminoglycans, which were fixed and stained on the electrophoresis strip with Alcian blue, was measured by cutting the strips and placing them in vials with a scintillation solution as described in our previous paper (Sobue *et al.*, 1978). Further identification of GAG components was performed enzymatically. Each solution containing 50 nmol of uronic acid was digested with chondroitinase ABC, chondroitinase AC and hyaluronate lyase, respectively, under the optimal conditions described in the indicated literature Yamagata *et al.*, 1968; Hiyama & Okada, 1975; Ohya & Kaneko, 1970). The enzyme-treated and untreated materials were submitted to cellulose-acetate membrane electrophoresis (see above).

Malignant neoplastic tissue used for analysis was the well-differentiated liposarcoma, composed of the mature fat cells with wide areas of myxoid tissue. In the myxomatous matrix, stained profusely

FIG. 1.—Microscopic section of liposarcoma tissue (Case 1) showing a myxomatous pattern (Alcian blue and H & E. ×240).

with Alcian blue, the stellate and fusiform cells were arranged into a loose network, as shown in Fig. 1. In some areas, a few rounded lipoblasts and bizarre lipoblasts with an abnormal nucleus were seen. In an autoradiograph, diffuse $^{35}$S-radioactivity was observed in the tumour cells of the myxomatous areas, as shown in Fig. 2, and the amount of $^{35}SO_4$ incorporated in the vascular cells was relatively large. Diffuse and low $^{3}$H-radioactivity was also seen in the tumour cells. In the case of lipoma consisting of mature lipocytes, the level of $^{3}$H-radioactivity observed in the tumour cells was not so high, and the amount of $^{35}$S labelling was significant in the vascular cells (Fig. 3). In the non-neoplastic adipose tissues, $^{35}$S labelling was relatively low.

FIG. 2.—Autoradiograph of section of liposarcoma (Case 1). $^{35}$S label is seen diffusely in the tumour tissue (Alcian blue and H & E. ×680).

The incorporation of $^{3}$H label into hyaluronic acid (ct/min/mg of defatted tissue) and that of $^{3}$H and $^{35}$S label into sulphated GAGs in each tissue is shown in the Table. Hyaluronic acid synthesis by the sarcoma tissue was 50–200 times that of the lipoma or the non-neoplastic adipose tissue. The GAG content of the sarcoma tissue was 10–30 times that of lipomas and adipose tissues. However,

FIG. 3.—Autoradiograph of section of lipoma (Case 4). $^{35}$S label is mainly in the vascular component (Alcian blue and H & E. ×480).

differences in $^{35}$S-sulphated GAG synthesis between the sarcomatous tissue, the lipomas and the adipose tissues were not so significant, though the activity was higher in the neoplastic tissues. $^{35}$S-dermatan sulphate synthesis was in most cases considerably higher than $^{35}$S-chondroitin sulphate synthesis.

The results of the autoradiography suggested that the amount of sulphated GAGs synthesized by the adipose tissues may relate to the amount of the vascular components, which were diffusely distributed in these tissues, whereas most of the hyaluronic acid detected was synthesized by the lipoblasts. There are two theories concerning the histogenesis of adipose tissue: (1) Adipose cells are merely fibroblasts which have accumulated excess lipid. (2) Adipose tissue develops from lipoblasts which became separated from mesenchymal cell early in embryonic life. It is well known that sulphated-GAG synthesis is one of differentiation of mesenchymal cells (Lovell *et al.*, 1966; Prodi & Romeo, 1967), and it was noted that hyaluronic acid is usually synthesized by fibroblastic cells only during the actively growing phase (Morris, 1960; Davidson, 1963). The present result, showing that hyaluronic acid production has a close relation to proliferation of lipoblasts, seems to indicate that the biological characteristic of lipoblasts and fibroblasts are similar in terms of GAG synthesis. Recently, in our laboratory, we observed GAG synthesis by subcutaneous mesenchymal tissues of SMA mice, by the procedure described in the present study. The interscapular brown fat tissue revealed a high level of GAG-synthesis. The amount of $^{3}H$ label incorporated into the GAG of brown fat (ct/min/mg dry tissue) was 1·4–3·0-fold higher than that of the subcutaneous loose connective tissues.

It was reported that hyaluronate, a major component of extracellular matrices through which cells migrate during embryonic tissue development and in regeneration, is also concentrated in the environment through which neoplastic cells invade local host tissues (Toole *et al.*, 1979). It was demonstrated in our previous studies that sulphated GAG, as well as hyaluronic acid, support the viability of tumour cells *in vivo* and *in vitro* (Takeuchi, 1966, 1972; Takeuchi *et al.*, 1974). It is conceivable that in the tumour presented here the growth of tumour cells is facilitated by GAG produced by the tumour cells themselves.

### REFERENCES

BITTER, T. & MUIR, H. M. (1962) A modified uronic acid carbazole reaction. *Anal Biochem.*, **4**, 330.

DAVIDSON, E. H. (1963) Heritability and control of differentiated function in cultured cells. *J. Gen. Physiol.*, **46**, 983.

FILIPE, M. I. & MACKENZIE, D. H. (1976) Histochemical characteristics of mucosubstances in three soft tissue tumours. *J. Pathol.*, **118**, 17.

HIYAMA, K. & OKADA, S. (1975) Crystallization and some properties of chondroitinase from *Arthrobacter aurescens*. *J. Biol. Chem.*, **250**, 1824.

KANEKO, M. (1974) Quantitative and qualitative analyses of mucopolysaccharides in lipoblastic tumors and rhabdomyosarcoma. *Connective Tissue*, **6**, 127.

KINDBLOM, L.-G. & ANGERVALL, L. (1975) Histochemical characterization of mucosubstances in bone and soft tissue tumors. *Cancer*, **36**, 985.

LOVELL, D., SCHORAH, C. J. & CURRAN, R. C. (1966) Glycosaminoglycans (acid mucopolysaccharides) in experimental granulomata. *Br. J. Exp. Pathol.*, **47**, 232.

MEYER, K. (1955) The chemistry of the mesodermal ground substances. *Harvey lect.*, **51**, 88.

MORRIS, C. C. (1960) Quantitative studies on the production of acid mucopolysaccharides by replicate cell cultures of rat fibroblasts. *Ann. N.Y. Acad. Sci.*, **86**, 876.

OHYA, T. & KANEKO, Y. (1970) Novel hyaluronidase from Streptomyces. *Biochim. Biophys. Acta*, **198**, 607.

PRODI, G. & ROMEO, G. (1967) The evolution of acid mucopolysaccharides in carrageenin granulomata. *Br. J. Exp. Pathol.*, **48**, 40.

SOBUE, M., TAKEUCHI, J., ITO, K., KIMATA, K. & SUZUKI, S. (1978) Effect of environmental sulfate concentration on the synthesis of low and high sulfated chondroitin sulfates by chick embryo cartilage. *J. Biol. Chem.*, **253**, 6190.

SOBUE, M., TAKEUCHI, J., MIURA, K., KAWASE, K., MIZUNO, F. & SATO, E. (1980) Glycosaminoglycan content and synthesis in gastric carcinoma. *Br. J. Cancer*, **42**, 78.

TAKEUCHI, J. (1966) Growth-promoting effect of acid mucopolysaccharides on Ehrlich ascites tumor. *Cancer Res.*, **26**, 797.

TAKEUCHI, J. (1972) Effect of chondroitinases on the growth of solid Ehrlich ascites tumour. *Br. J. Cancer*, **26**, 115.

TAKEUCHI, J., SOBUE, M., YOSHIDA, M. & SATO, E. (1978) Glycosaminoglycan-synthetic activity of pleomorphic adenoma, adenoid cystic carcinoma and non-neoplastic tubuloacinar cells of the salivary gland. *Cancer*, **42**, 202.

TAKEUCHI, J., TCHAO, R. & LEIGHTON, J. (1974) Protective action of mucopolysaccharides on dog kidney cell line MDCK in meniscus-gradient culture. *Cancer Res.*, **34**, 161.

TOOLE, B. P., BISWAS, C. & GROSS, J. (1979) Hyaluronate and invasiveness of rabbit V2 carcinoma. *Proc. Natl Acad. Sci. U.S.A.*, **76**, 6299.

WINSLOW, D. J. & ENZINGER, F. M. (1960) Hyaluronidase-sensitive acid mucopolysaccharides in liposarcomas. *Am. J. Pathol.*, **37**, 497.

YAMAGATA, T., SAITO, H., HABUCHI, O. & SUZUKI, S. (1968) Purification and properties of bacterial chondroitinases and chondrosulfatases. *J. Biol. Chem.*, **243**, 1523.


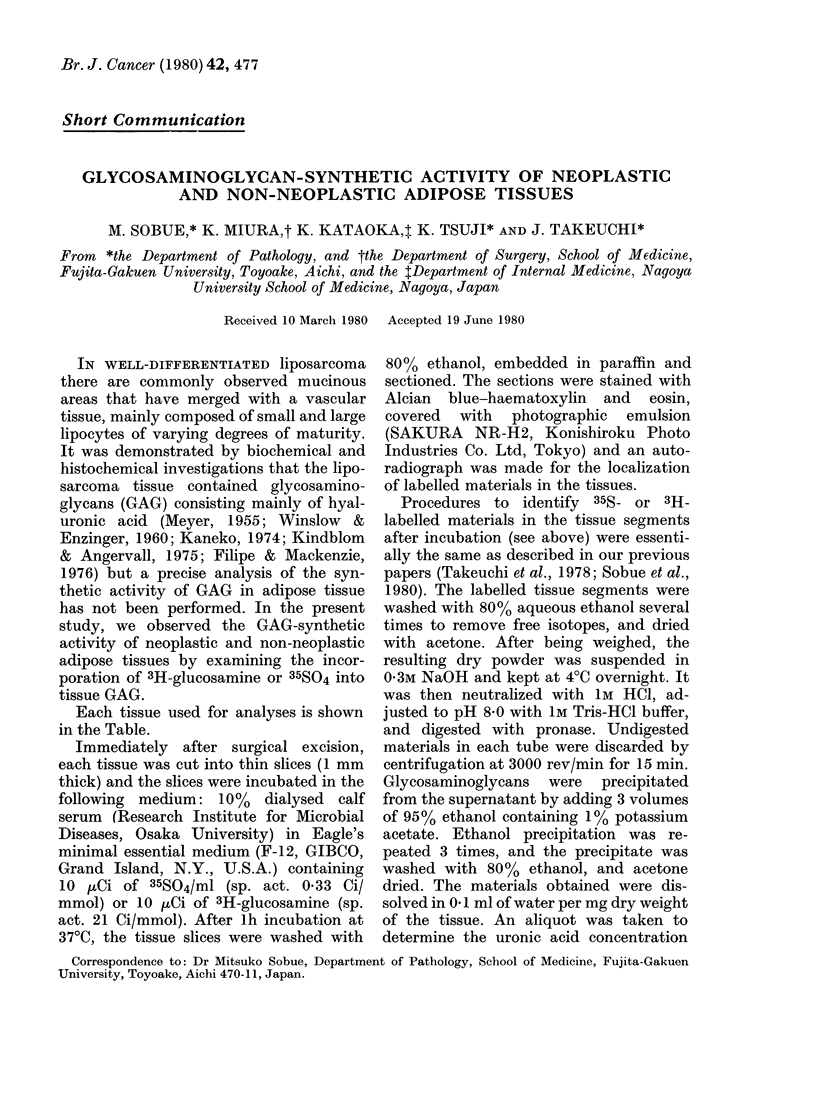

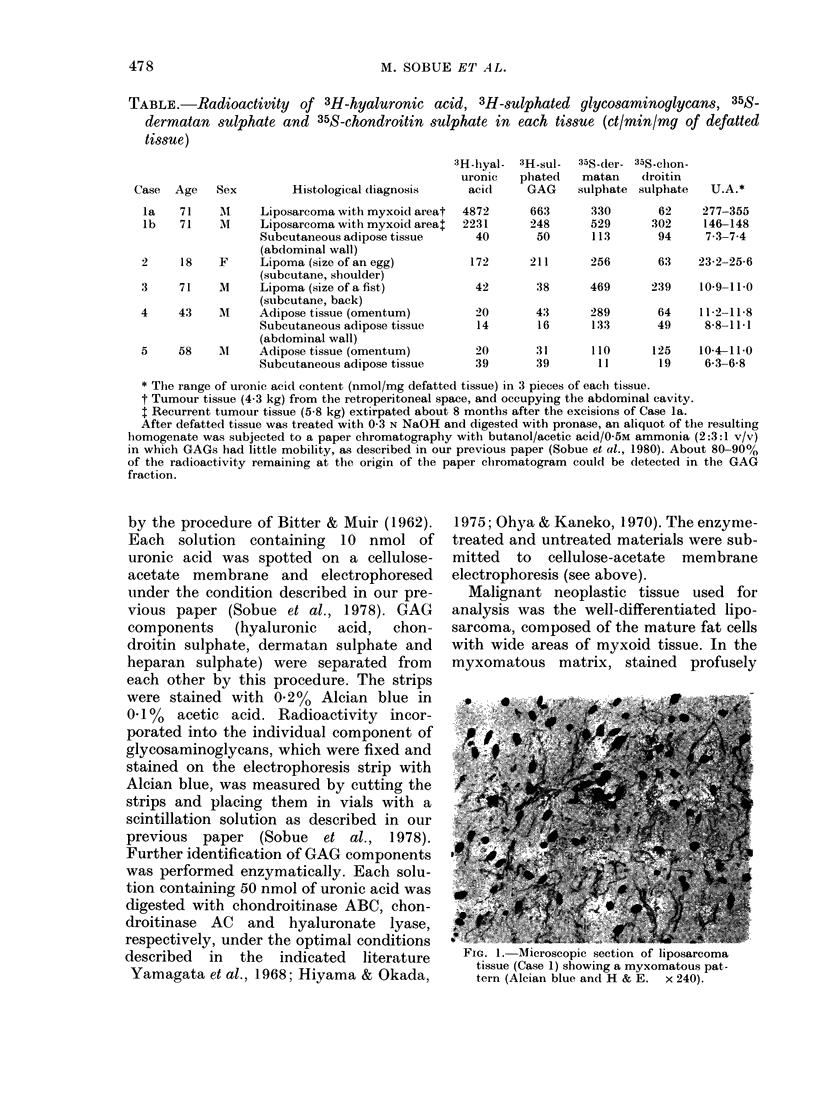

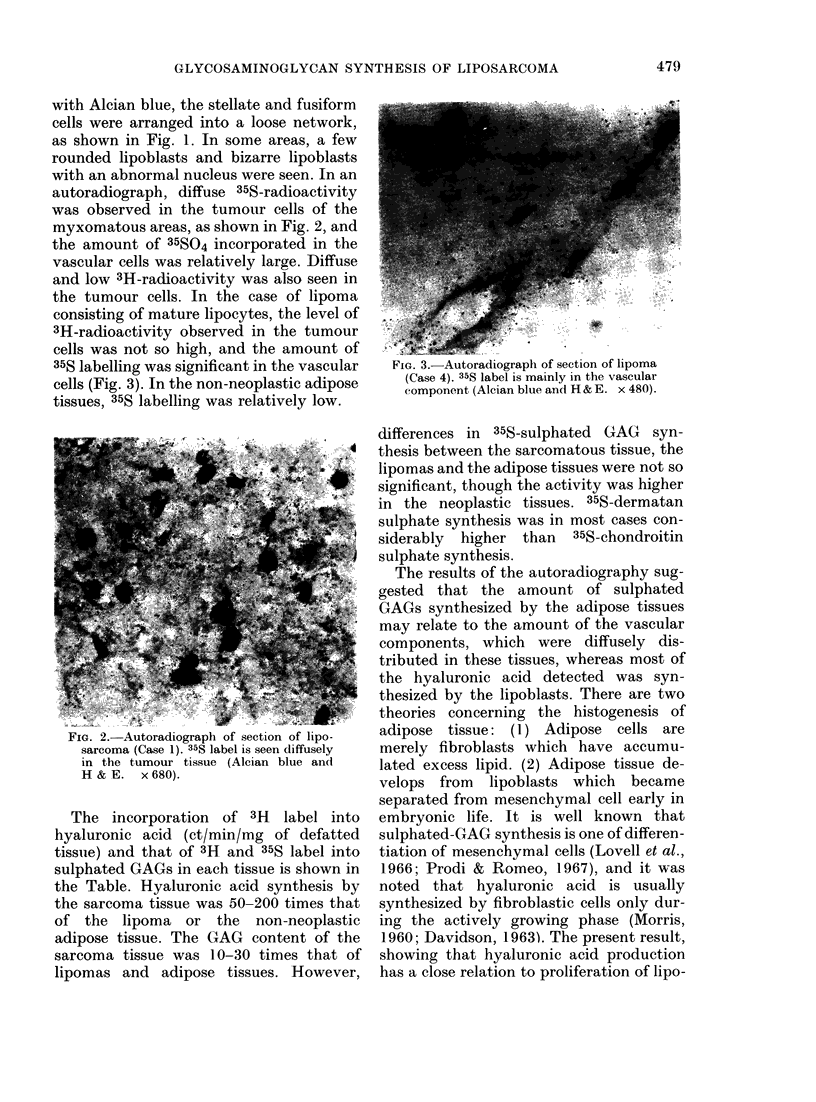

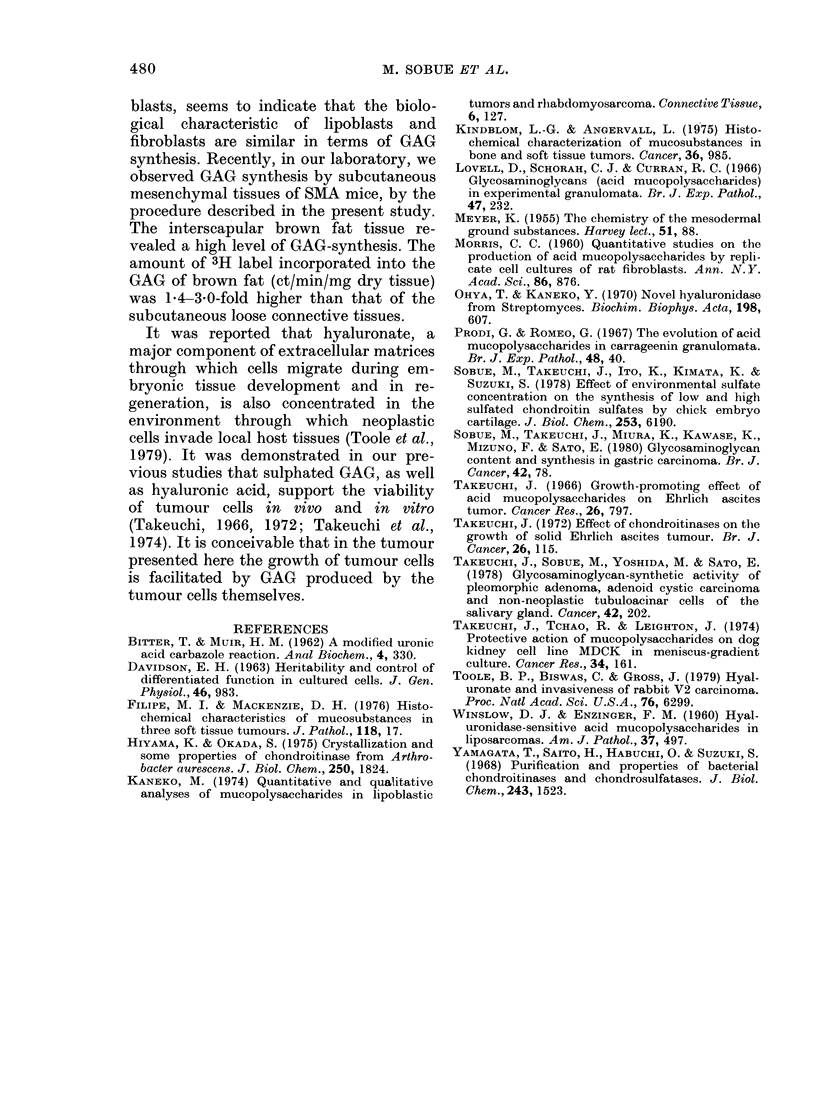


## References

[OCR_00347] BITTER T., MUIR H. M. (1962). A modified uronic acid carbazole reaction.. Anal Biochem.

[OCR_00351] DAVIDSON E. H. (1963). Heritability and control of differentiated function in cultured cells.. J Gen Physiol.

[OCR_00356] Filipe M. I., Mackenzie D. H. (1976). Histochemical characteristics of muco-substances in three soft-tissue tumours.. J Pathol.

[OCR_00361] Hiyama K., Okada S. (1975). Crystallization and some properties of chondroitinase from Arthrobacter aurescens.. J Biol Chem.

[OCR_00373] Kindblom L. G., Angervall L. (1975). Histochemical characterization of mucosubstances in bone and soft tissue-tumors.. Cancer.

[OCR_00384] MEYER K. (1955). The chemistry of the mesodermal ground substances.. Harvey Lect.

[OCR_00394] Ohya T., Kaneko Y. (1970). Novel hyaluronidase from streptomyces.. Biochim Biophys Acta.

[OCR_00399] Prodi G., Romeo G. (1967). The evolution of acid mucopolysaccharides in carrageenin granulomata.. Br J Exp Pathol.

[OCR_00404] Sobue M., Takeuchi J., Ito K., Kimata K., Suzuki S. (1978). Effect of environmental sulfate concentration on the synthesis of low and high sulfated chondroitin sulfates by chick embryo cartilage.. J Biol Chem.

[OCR_00411] Sobue M., Takeuchi J., Miura K., Kawase K., Mizuno F., Sato E. (1980). Glycosaminoglycan content and synthesis in gastric carcinoma.. Br J Cancer.

[OCR_00422] Takeuchi J. (1972). Effect of chondroitinases on the growth of solid Ehrlich ascites tumour.. Br J Cancer.

[OCR_00417] Takeuchi J. (1966). Growth-promoting effect of acid mucopolysaccharides on Ehrlich ascites tumor.. Cancer Res.

[OCR_00427] Takeuchi J., Sobue M., Yoshida M., Sato E. (1978). Glycosaminoglycan-synthetic activity of pleomorphic adenoma. Adenoid cystic carcinoma and nonneoplastic tubuloacinar cells of the salivary gland.. Cancer.

[OCR_00434] Takeuchi J., Tchao R., Leighton J. (1974). Protective action of mucopolysaccharides on dog kidney cell line MDCK in meniscus-gradient culture.. Cancer Res.

[OCR_00440] Toole B. P., Biswas C., Gross J. (1979). Hyaluronate and invasiveness of the rabbit V2 carcinoma.. Proc Natl Acad Sci U S A.

[OCR_00445] WINSLOW D. J., ENZINGER F. M. (1960). Hyaluronidase-sensitive acid mucopolysaccharides in liposarcomas.. Am J Pathol.

[OCR_00450] Yamagata T., Saito H., Habuchi O., Suzuki S. (1968). Purification and properties of bacterial chondroitinases and chondrosulfatases.. J Biol Chem.

